# Distinguishing time-delayed causal interactions using convergent cross mapping

**DOI:** 10.1038/srep14750

**Published:** 2015-10-05

**Authors:** Hao Ye, Ethan R. Deyle, Luis J. Gilarranz, George Sugihara

**Affiliations:** 1 Scripps Institution of Oceanography, University of California San Diego, La Jolla, CA, USA; 2 Integrative Ecology Group, Estación Biológica de Doñana, CSIC, Sevilla, Spain

## Abstract

An important problem across many scientific fields is the identification of causal effects from observational data alone. Recent methods (convergent cross mapping, CCM) have made substantial progress on this problem by applying the idea of nonlinear attractor reconstruction to time series data. Here, we expand upon the technique of CCM by explicitly considering time lags. Applying this extended method to representative examples (model simulations, a laboratory predator-prey experiment, temperature and greenhouse gas reconstructions from the Vostok ice core, and long-term ecological time series collected in the Southern California Bight), we demonstrate the ability to identify different time-delayed interactions, distinguish between synchrony induced by strong unidirectional-forcing and true bidirectional causality, and resolve transitive causal chains.

A fundamental question in science is identifying the causal relationships between variables. The conventional approach to this problem is to observe the outcomes of controlled experiments; however, this is not always possible due to moral, legal, or feasibility reasons. Consequently, the ability to infer causality using only observational data is a highly valuable tool with applications in many fields of study [e.g., financial systems, ecosystems, neuroscience^1^,^2^,^3^,^4^].

Early on, Bishop Berkeley^5^ warned that the co-occurrence of events did not necessarily mean that they are causally related (i.e., correlation does not imply causation). Even so, the use of correlation to suggest causality (or more frequently, the lack of correlation suggesting no causality) has remained a common, heuristic notion, and is still commonly applied today. In 1969, however, Granger^1^ suggested an alternative framework for detecting causality based on the idea of using prediction as a criterion. In the Granger causality framework, a variable *x* is said to “cause” variable *y* if *x* has unique information (i.e., not found in other variables) that can improve the prediction of *y*. Thus, causality could be inferred if the optimal model for *y* improves when *x* is included. However, Granger noted that this approach might not apply in dynamic systems, and indeed, Sugihara *et al.*^4^ showed that it does not: in dynamic systems with behaviors that are at least somewhat deterministic, information about past states is carried forward through time (i.e., the system is not completely stochastic). Thus, Takens’ Theorem^6^ applies, and so if *x* is indeed causal to *y*, then information about *x* must be recorded in *y*. Consequently, causal variables (i.e., *x*) cannot contain unique information (it will also be recorded in the affected variables), and so Granger’s test is invalid (except in certain cases; see Discussion).

As an alternative test for causality, Sugihara *et al.*^4^ suggested a new method, convergent cross mapping (CCM). It follows from Takens’ Theorem^6^ that if *x* does influence *y*, then the historical values of *x* can be recovered from variable *y* alone. In practical terms, this is accomplished using the technique of “cross mapping”: a time delay embedding is constructed from the time series of *y*, and the ability to estimate the values of *x* from this embedding quantifies how much information about *x* has been encoded into *y*. Thus, the causal effect of *x* on *y* is determined by how well *y* cross maps *x*. This approach is described in further detail in the materials and methods, but also summarized in this short instructional animation: https://www.youtube.com/playlist?list=PL-SSmlAMhY3bnogGTe2tf7hpWpl508pZZ.

Although CCM can be successfully applied to systems with weak to moderate coupling strengths, Sugihara *et al.* observed that exceptionally strong unidirectional forcing can lead to the phenomenon of “generalized synchrony”^7^. In these situations, the dynamics of a response variable, *y*, become dominated by those of the driving variable, *x*, such that the full system (consisting of both the response variable and driving variable) collapses to just that of the driving variable. Although there is no causal effect of *y* on *x*, the states of the driving variable *x* can uniquely determine the response variable *y*, and so CCM is observed in both directions (i.e., *x* cross maps *y* and *y* cross maps *x*). Thus, CCM appears to be limited by the fact that it may not be able to distinguish between bidirectional causality and strong unidirectional causality that leads to synchrony.

Here, we propose an extension to CCM that can resolve this problem: by explicitly considering different lags for cross mapping, it is possible to determine whether a driving variable acts with some time delay on a response variable. In the case of synchrony caused by strong unidirectional forcing, this approach should detect a negative lag for cross mapping in the true causal direction (the response variable is better at predicting the past values of the driving variable rather than future values) and a positive lag in the other direction (the driving variable best predicts the future response). Thus, this “asynchrony” reflecting the time lag in the response can be used to distinguish between bidirectional causality and generalized synchrony when there is a detectable lag in the response time between causes and effects.

This extension of CCM has several additional applications: the identification of time delays in causation can be informative, for instance in understanding delays in interventions or manipulations. It can also be used to identify the causal effects of stochastic drivers that have no dynamics (for which general cross mapping may not succeed), and can even correctly determine the order of variables in a transitive causal chain.

## Results and Discussion

### Model Simulations

**Figure 1:**
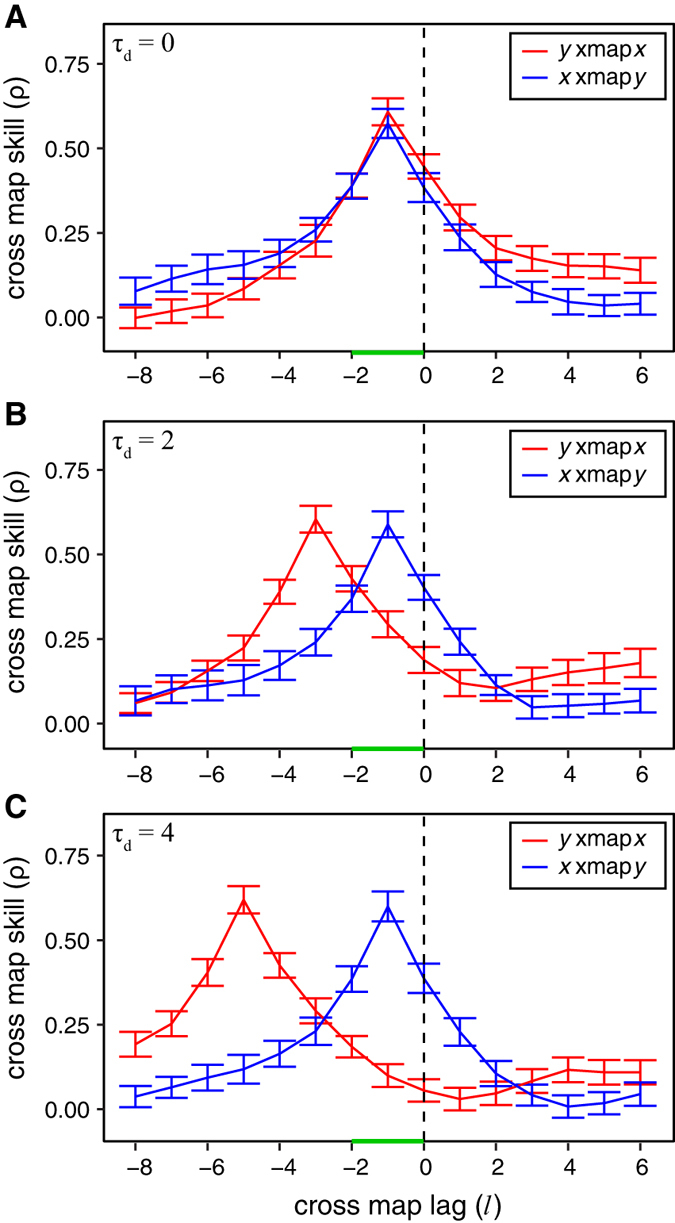
Model demonstration of causal lags and optimal cross mapping using a 2-species logistic model with bidirectional forcing. Cross-mapping skill (ρ) is shown as a function of cross-mapping lag for three different time delays, τ_d_, in the effect of *x* on *y*. Here, “*y* xmap *x*” refers to using *y* and its lags to cross map variable *x* with time lag *l*. (**A**) With τ_d_ = 0, both variables respond to each other within a single time-step (*y*(*t* + 1) is influenced by *x*(*t*) and vice-versa), and so the optimal cross map lag occurs at *l* = −1, falling within the embedding vector (green bar) as expected. (**B**,**C**) For τ_d_ = 2 or 4, the effect of *x* on *y* is delayed, and so the optimal lag for *y* cross mapping *x* (i.e., red line, measuring the effect of *x* on *y*) shifts back by a corresponding amount, while *x* cross mapping *y* is unchanged. Plots show mean cross map skill and standard deviation over 100 random libraries (see Materials and Methods).

Figure 1 shows the results of extended CCM applied to the two-species coupled logistic map (equation (1)). As shown in the first panel (Fig. 1A), where causation occurs with an effective delay of 1 time step (*y*(*t*) affects *x*(*t* + 1) and vice-versa), the optimal cross mapping in both directions occurs at a lag of −1. Moreover, as expected, a time delay in the effect of *x* on *y* (Fig. 1B,C), produces optimal cross mapping (from *y* to *x*) with a lag corresponding to the degree of time delay. Extending this analysis to systems with random coefficients (see Supplementary Information), the result is robust, with only a few outliers that exhibit optimal cross mapping at different lags (Figure S1). This validates a basic rule of thumb for bidirectional causality: we may reasonably expect optimal cross mapping lags to be negative, and with the magnitude of the lag roughly equal to the time delay of causality.

**Figure 2:**
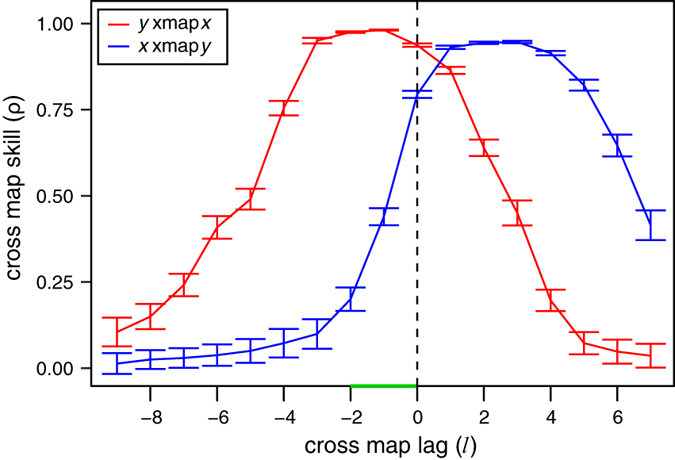
Generalized synchrony in a 2-species logistic model with unidirectional forcing. In this system, the dynamics of *y* becomes enslaved to *x*, and so *y* can be predicted from *x*. Since *x* affects future values of *y*, *x* is best able to cross map *y* forward in time (*l* ~ 3 > 0), whereas cross mapping in the true direction shows optimal prediction for negative time lags (*l* ~ −1 < 0, as in Fig. 1). Thus, even though there is cross mapping in both directions, we can use the positive optimal prediction lag to distinguish the direction of causality. As in Fig. 1, “y xmap *x*” refers to using *y* and its lags to cross map variable *x* with time lag *l*; plots show mean cross map skill and standard deviation over 100 random libraries (see Materials and Methods).

For systems where strong unidirectional causality leads to generalized synchrony (equation (2)), a time delay in the response can be detected using extended convergent cross mapping. Although the response variable “synchronizes” to the causal variable, if causality is not instantaneous, the synchronization occurs with some lag that can then be identified using extended convergent cross mapping. In Fig. 2, we find that the optimal cross map lag from *y* to *x* is negative, as expected; *x* causes *y*, and so cross map skill is better when estimating the historical influence of *x* from the response variable *y*. Conversely, the optimal cross map lag from *x* to *y* is positive, because even with synchrony, there is no flow of causal information from *y* to *x*, and so changes in *x* are not reflected in *y* until sometime in the future. Thus, the positive lag from *x* cross mapping *y* informs us that there is unidirectional causality, even when the interaction is strong enough to result in synchrony. Again, extending this analysis to similar systems with random coefficients (see Supplementary Information), we find that optimal cross map lags can reliably distinguish between generalized synchrony and bidirectional causality (Figure S2).

**Figure 3:**
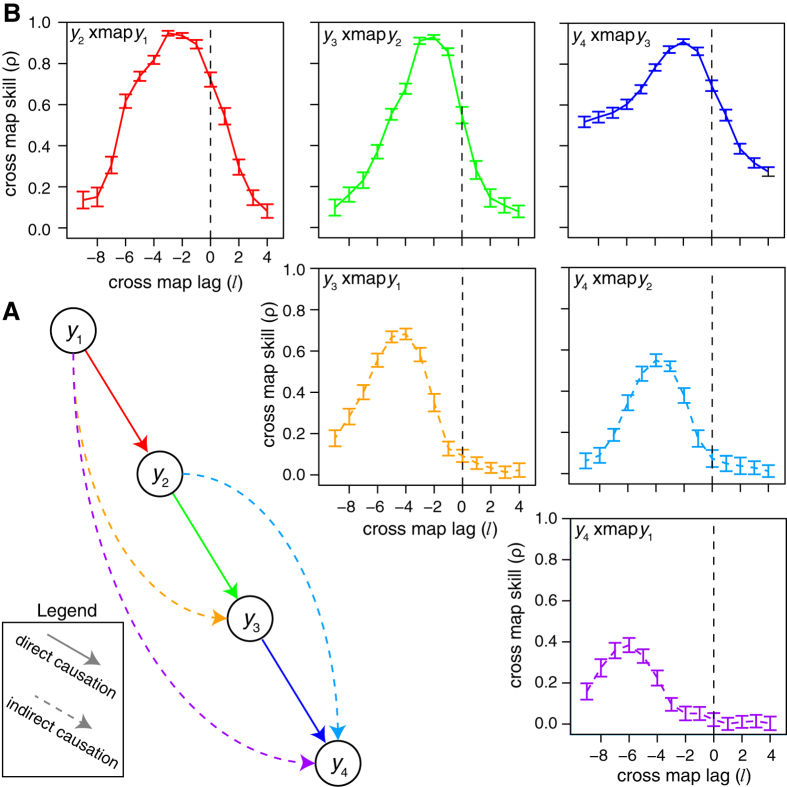
Direct and indirect causality in a transitive causal chain. (**A**) In this system, *y*_1_ causes *y*_2_ causes *y*_3_ causes *y*_4_ such that indirect causation from *y*_1_ to *y*_3_, *y*_2_ to *y*_4_, and *y*_1_ to *y*_4_ occurs. (**B**) Using extended CCM, the direct links (top row) are strongest with the highest cross map skill and the most immediate effects (*l* ~ −2), the indirect links separated by one node (middle row) have moderate cross map skill and somewhat delayed effects (*l* ~ −4), and the indirect link from *y*_1_ to *y*_4_ (bottom row) is the weakest and with the longest time delay (*l* ~ −6). Here “*y*_*i*_ xmap *y*_*j*_” refers to using *y*_*i*_ and its lags to cross map to *y*_*j*_. Plots show mean cross map skill and standard deviation over 100 random libraries (see Materials and Methods).

As discussed by Sugihara *et al.*^4^, CCM can detect indirect causality that occurs through a transitive causal chain. For example, in the system depicted in Fig. 3A, *y*_1_ causes *y*_2_ causes *y*_3_ causes *y*_4_ (equation (3)). With CCM, we can detect these direct causal connections (e.g., using the cross map from *y*_j_ to *y*_i_ to infer the effect of *y*_i_ on *y*_j_). However, there are also indirect effects from *y*_1_ to *y*_3_, *y*_2_ to *y*_4_, and *y*_1_ to *y*_4_. These indirect effects may also appear significant in CCM if coupling is strong enough. To unravel the direct from indirect effects in this system, we can apply extended CCM to identify the optimal cross map lags and optimal cross map skill (Fig. 3B). For the direct links (top row of Fig. 3B), optimal cross mapping occurs with high skill and a small negative lag (*l* ~ −2); for indirect links separated by a single node (middle row of Fig. 3B), optimal cross mapping occurs with moderate skill and a moderate negative lag (*l* ~ −4); and for the indirect link from *y*_1_ to *y*_4_ (separated by both *y*_2_ and *y*_3_), optimal cross mapping is weak, and at a large negative lag (*l* ~ −6). When this analysis was repeated for model systems with random coefficients (see Supplementary Information), the differences in optimal cross map lag were relatively robust (Figure S3). However, cross map skill showed more variance, suggesting that it is a less reliable indicator of direct vs. indirect causation. The outliers are likely a result of stable dynamics (with cross map skill, ρ that reaches 1), since this is a simple model simulated without process error.

### Veilleux’s Paramecium-Didinium Experiment

**Figure 4:**
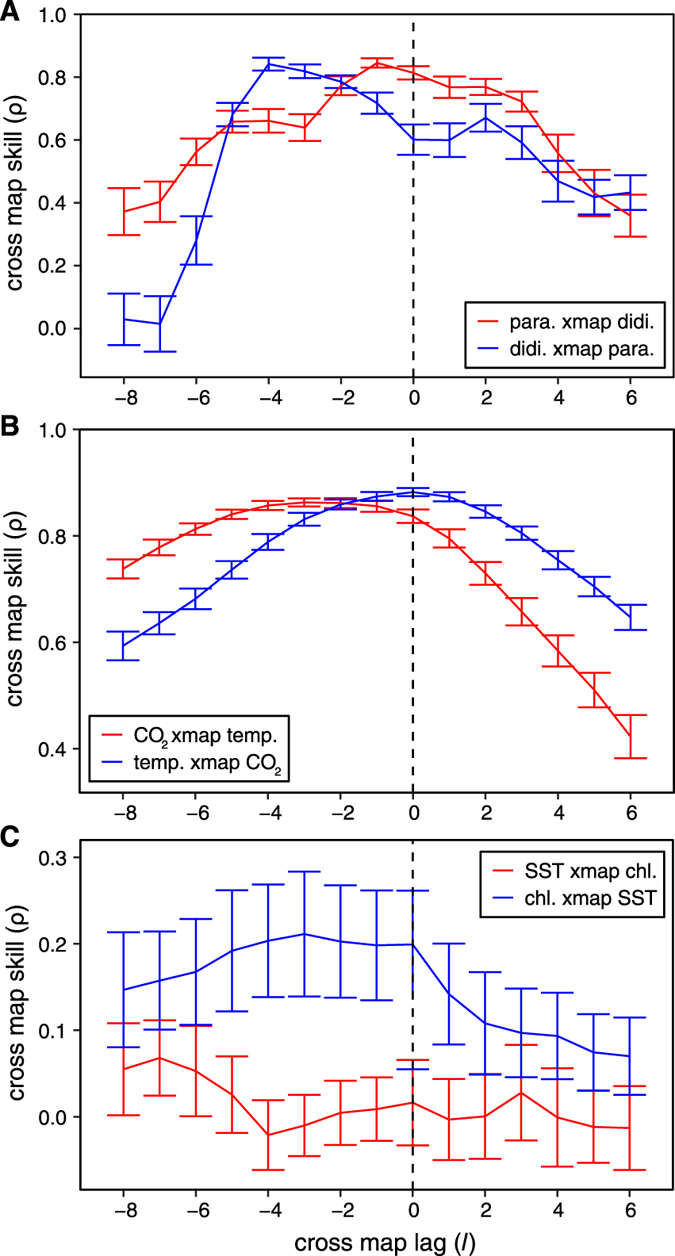
Applying extended CCM to real world examples. (**A**) Extended CCM analysis of time series from Veilleux’s predator-prey experiment^8^ with *Paramecium aurelia* (prey) and *Didinium nasutum* (predator) reveals bidirectional causality. While the effect of predators on prey (red, “ para. xmap didi.”) is immediate, the effect of prey on predators (blue, “didi. xmap para.”) shows a distinct lag, as prey ingestion does not instantaneously translate into population growth. (**B**) Analysis of causality between Earth atmospheric CO_2_ and temperature using time series data from the Vostok ice core for the previous 412,000 years. As expected CO_2_ has a nearly instantaneous effect on temperature (blue, “temp. xmap CO_2_”) due to the fast-acting greenhouse gas effect, while the influence of temperature on CO_2_ is much slower, with an optimal CCM lag of ~3000 years (red, “CO_2_ xmap temp.”). (**C**) Analysis of weekly averages of sea surface temperature (SST) and chlorophyll-a at SIO pier in La Jolla, CA suggests that the effect of SST occurs with a lag of 1–4 weeks (blue, “chl. xmap SST”). All plots show mean cross map skill and standard deviation over 100 random libraries (see Materials and Methods).

Applying extended CCM to the time series of *Paramecium* and *Didinium* from Veilleux’s lab experiments^8^, we confirm the results of Sugihara *et al.*^4^ showing bidirectional causality. However, whereas Sugihara *et al.* suggested that the difference in cross mapping predictability (with a lag of 0) was indicative of stronger top-down forcing, our analysis here reveals another layer to the story: considering different lags, we find that cross mapping predictability is roughly equal at optimal lag values (Fig. 4A), suggesting that top-down and bottom-up effects are equally important. We do note that the optimal cross mapping lag does depend on the interaction: an optimal lag of −1 for the *Paramecium* cross mapping *Didinium* direction suggests that *Paramecium* respond quickly to changes in *Didinium* abundance. However, an optimal lag of −4 for the *Didinium* cross mapping *Paramecium* direction suggests that *Didinium* respond more slowly to changes in *Paramecium* abundance. These results are consistent with the ecological context of this system^9^: the prey (*Paramecium*) respond quickly to predators (*Didinium*) because predator-induced mortality has an immediate (negative) effect on the abundance of prey, whereas the abundance of predators (*Didinium*) responds more slowly to prey (*Paramecium*), because of the time delay in converting food into new individuals.

### Vostok Ice Core

Figure 4B shows the application of extended convergent cross mapping to time series of CO_2_ and temperature reconstructed from the Vostok ice core^10^. Here, we detect bidirectional causality (the optimal cross mapping lag is negative in both directions), suggesting that there is a positive feedback in the Earth’s climate system between temperature and greenhouse gases. Notably, the optimal lag in the temperature to CO_2_ direction matches current scientific knowledge that greenhouse gases have a rapid effect on temperature (faster than the 1000-year timescale of the data), while the influence of global temperature on greenhouse gases likely occurs through slower mechanisms (e.g., increased plant respiration at higher temperatures^11^, release of greenhouse gases from terrestrial^12^ or marine ecosystems^13^). A detailed analysis of this system appears in van Nes *et al.*^14^.

### Southern California Bight

In Figure 4C, we show the results of extended CCM applied to long-term time series of chlorophyll-a and sea surface temperature measured at the Scripps Institution of Oceanography pier. As expected, there is no effect of chlorophyll-a on SST (red line). However, we do identify a causal influence of SST on chlorophyll-a, suggesting that the physical environment plays a role in determining phytoplankton abundances (which are proxied by concentrations of chlorophyll-a). Moreover, optimal cross mapping occurs with a lag of 3 weeks, suggesting that the physical drivers of algae populations act with a lag of several weeks. Ideally, if other causal drivers show similar time delays in their effects, then it may be possible to produce models that can forecast events such as algal blooms several weeks in advance!

### Stochastic Drivers

We note that in certain systems, especially those with stochastic drivers that contain unique information, Granger causality may correctly identify causal interactions. Indeed, Granger causality has been successful when applied to system consisting solely of stochastic components. However, in situations where both cause and effect have deterministic dynamics, causal information cannot be isolated from amongst the affected variables, and alternative methods, such as CCM must therefore be used.

### Final Remarks

Here, we have shown that explicitly considering time lags when applying convergent cross mapping can be a valuable tool beyond the simple test of whether two variables are causally related. Although this general approach has been explored elsewhere^15^, here we show how the CCM framework can be directly extended to account for temporal delays. As demonstrated in our model simulations, CCM can now distinguish synchrony induced by strong unidirectional forcing from true bidirectional causation (Fig. 2), as well as order nodes in transitive causal chains that produce direct and indirect causal links (Fig. 3).

In addition, we show how identification of time delays can clarify our understanding of the causal effects, which can be valuable in producing a more detailed and mechanistic description of causal dynamics in real systems. For example, knowing the approximate time delay of causal interactions can be important when forecasting future events – although in general, a single time series contains all necessary dynamic information, this will not be the case when stochastic drivers are influencing the dynamics. Since the stochastic driver has unique information, it must be explicitly included at the appropriate lag for optimal predictability (see ref. 16, 17 for examples). Moreover, understanding the delayed effect of external drivers will be important in management scenarios, as knowing when to expect the system to respond to interventions or manipulations will guide future management actions.

## Methods

### Convergent Cross Mapping

The basic principle of cross mapping involves reconstructing system states from two time series variables and then quantifying the correspondence between them using nearest neighbor forecasting^18^. Reconstruction is done using the method of time delay embedding: with the system state represented using successive lags of a single time series^6^,^19^. For example, given a time series {*y*(*t*)}, an *E*-dimensional reconstruction uses *E* successive lags of *y*, each separated by a time step *τ*: < *y*(*t*), *y*(*t* - *τ*), … *y*(*t* - (*E*-1)*τ*) >.

We note that the optimal value of the embedding dimension *E* depends on several factors, including system complexity, time series length, and noise. In the case of model systems, the number of interacting variables is known exactly and was used to select *E*. In the remaining cases, the value of *E* was determined empirically by applying simplex projection^18^ to the individual time series and choosing the optimal *E*. Since most time series were not overly sampled in time, we fixed τ = 1 for all systems.

In the case of a system where *x* causes *y*, Takens’ Theorem^6^ implies that there should be a correspondence between the state ***y***(*t*) and the contemporaneous state ***x***(*t*). Convergent cross mapping (CCM^4^) quantifies this relationship using simplex projection (a nearest-neighbor forecasting method, see ref. 18 for details) to estimate the scalar value *x*(*t*) from the reconstructed vector ***y***(*t*) (see Movie S3 of ref. 4 for details). Although different performance metrics are possible, here we use Pearson’s correlation coefficient between the estimated and observed values of *x*(*t*) as an indicator of “cross map skill”.

We note that, in general, one may compute a function that maps from ***y***(*s*) to the entire vector as opposed to just the scalar value *x*(*s*)^20^,^21^. However, doing so can decrease the sensitivity of the cross mapping idea, because the errors are no longer scalar values, but *E*-dimensional vectors, for which common distance metrics can become meaningless^22^. Moreover, by estimating entire vectors, we limit the capability to use cross mapping to analyze time delays in the effect of *x* on *y*, which we show here can be informative in an extended version of CCM (see below).

### Extended Convergent Cross Mapping

**Figure 5:**
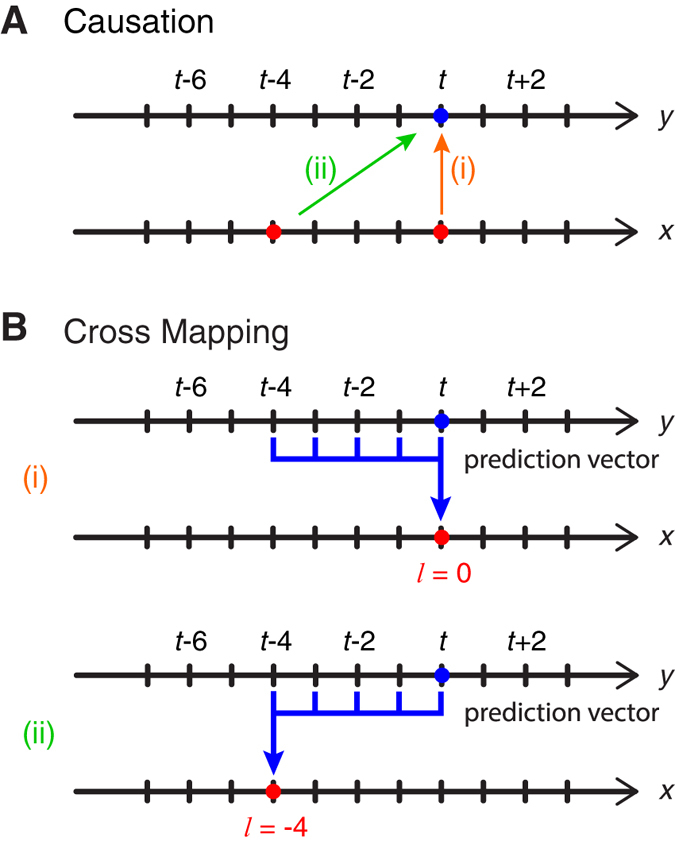
Effect of time delays on cross mapping. Panel (**A**) shows causation for two cases: (i) no time delay in the effect of *x* on *y* (i.e., *y* responds instantaneously to *x*), and (ii) *y* responds to *x* with a time delay of 4 (time steps). Panel (**B**) shows (i) cross mapping with *l* = 0, equivalent to the original formulation by Sugihara *et al.*^4^ and (ii) cross mapping with *l* = −4, which may be expected to be better than *l* = 0 when *x* acts on *y* with some time delay.

Standard cross mapping when *x* causes *y* (Fig. 5A) computes the predictability of *x*(*t*) from the *E*-dimensional reconstruction (Fig. 5B.i). However, the general theory of CCM^4^, based on generalizations of Takens’ Theorem^23^,^24^, suggests that we should also be able to cross map from ***y***(*t*) to *x*(*t* + *l*), for any reasonable lag value of *l*, since the variable *x*(*t* + *l*) is simply another observation function of the system. In fact, if *x* acts on *y* with some time delay (Fig. 5A.ii), then the current state of the system, ***y***(*t*), will better predict the past values of *x* (Fig. 5B.ii).

In general, we note that optimal predictability may be expected to occur for some *l* < 0, even if *y* responds instantaneously to *x*^25^. In other words, the state of the system at a time *t* is often best estimated from a reconstruction that includes both past and future values. This phenomenon occurs because information in a dynamic system can be thought of as propagating both forwards and backwards through time. In other words, knowing the exact value of variable *x* at time *t* restricts the likely set of possible futures (the value at time *t* + 1) as well as the likely set of possible pasts (the state at time *t* − 1). Furthermore, the exact amount of information contained in past (and future) values of *x* is determined by the rate at which predictability decreases when we forecast further into the future (or past). Consequently, this means the most information about the current system state occurs with a combination of forward and backward lags^25^: a time-centered embedding that balances positive and negative lags: <*y*(*t*), *y*(*t* − *τ*), *y*(*t* + *τ*), … *y*(*t* − (*E* − 1)*τ*/2), *y*(*t* + (*E* − 1)*τ*/2)>. In the context of extended CCM, this then suggests that the optimal lag will occur in the middle of the prediction vector: *l* = (*E* − 1) *τ/*2. In reality, however, the optimal lag will vary from system to system; so while the “middle of the vector” is a useful heuristic, optimal cross mapping at any lag that lies within the embedding vector, −(*E* − 1) *τ* ≤ *l* ≤ 0, is consistent with an influence of *x* on *y* with no time delay.

### Two-Species Model System with Bidirectional Causality

We first consider a simple model system consisting of 2 coupled logistic difference equations:


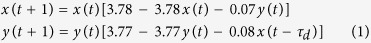


where *τ*_*d*_ is the time delay for the effect of *x* on *y*. The system is initialized as *x*(1) = 0.2 and *y*(1) = 0.4, and run for 3000 time steps, with different values for the time delay: *τ*_*d*_ = 0, *τ*_*d*_ = 2, and *τ*_*d*_ = 4. Using extended CCM, we analyze this system using *E* = 2, *τ* = 1, selecting 100 random libraries of 200 vectors over time points 101–2000, and computing cross map skill for time points 2001–3000.

### Two-Species Model System with Synchrony

We also examine a modified form of the above system with causality from *x* to *y* only:


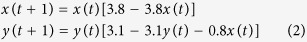


As above, the system is initialized as *x*(1) = 0.2 and *y*(1) = 0.4, and run for 1000 time steps. Because of the strong forcing of *x* on *y*, the dynamics of *y* are entrained to those of *x* [i.e., “generalized synchrony”^7^]. Thus, we apply extended CCM to identify the optimal cross map lag and distinguish this case from the case of bidirectional causality. In this system, we also use *E* = 2, *τ* = 1, selecting random libraries of 200 vectors over time points 101–2000, and computing cross map skill for time points 2001–3000.

### Four-Species Model System

To demonstrate extended CCM in systems with indirect causality (as a result of a transitive causal chain), we consider a 4-species model system. The system is initialized as *y*_1_(1) = *y*_2_(1) = *y*_3_(1) = *y*_4_(1) = 0.4, and evolves according to:


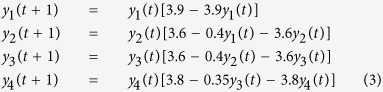


Although the only direct causal links are from *y*_1_ to *y*_2_, from *y*_2_ to *y*_3_, and from *y*_3_ to *y*_4_, this creates a transitive chain of causality, such that there is an indirect influence of *y*_1_ on *y*_3_, from *y*_2_ to *y*_4_, and from *y*_1_ to *y*_4_ (Fig. 3a). Thus, we apply extended CCM with *E* = 4 and *τ* = 1 to distinguish between direct and indirect causation. For each pair, we sample 100 random libraries of size 200 from time points 101–1000 and compute the cross map skill for time points 2001–3000.

### Paramecium-Didinium Predator-Prey System

We examine causality in a classical predator-prey system, the *Paramecium*-*Didinium* protozoan system using experimental time series from Veilleux^8^, who refined earlier work from Gause^26^ and Luckinbill^27^ to establish sustained oscillations. The data we used came from dataset 11a, and can be found at: http://robjhyndman.com/tsdldata/data/veilleux.dat. CCM analysis was done using *E* = 3, and *τ* = 1. Libraries were bootstrap samples over all 71 points of data, and cross map skill was computed using leave-one-out cross-validation over the same.

### Vostok Ice Core

Time series for historical Earth temperature and atmospheric CO_2_ concentration were based on reconstructions from the Vostok ice core^8^ and span ~410,000 years. To produce time series with regular intervals, we linearly interpolated the raw reconstructions to obtain estimates of temperature and CO_2_ spaced every 1000 years. CCM analysis was done by sampling 100 random libraries of size 100 and predicting over all 412 points of data, using leave-one-out cross-validation, *E* = 4, and *τ* = 1.

### Scripps Pier Time Series

Chlorophyll-a data came from measurements collected twice weekly at the end of the Scripps Institution of Oceanography’s pier (SIO Pier) as part of the Southern California Coastal Ocean Observing System, Harmful Algal Bloom Monitoring Program. Sea Surface Temperature (SST) was sampled daily as part of the Shore Stations Program, also at SIO Pier. Because of irregular sampling, we processed the data to construct weekly time series for the period June 30, 2008 to May 26, 2014. Extended CCM was then applied to investigate the relationship between SST and chlorophyll-a using *E* = 4 and *τ* = 1 (corresponding to 1 week) and sampling 100 random libraries of size 100 and predicting over all 306 points of data.

## Additional Information

**How to cite this article**: Ye, H. *et al.* Distinguishing time-delayed causal interactions using convergent cross mapping. *Sci. Rep.* **5**, 14750; doi: 10.1038/srep14750 (2015).

## Supplementary Material

Supplementary Information
