# Supplementary material for: Distinguishing time-delayed causal interactions using convergent cross mapping
Source: Sci Rep. 2015 Oct 5;5:14750. doi: 10.1038/srep14750 (PMC4592974; doi:10.1038/srep14750)
Supplement: Supplementary Information [file srep14750-s1.pdf]

Supplementary Information for

Distinguishing time-delayed causal interactions using convergent cross mapping

Hao Ye[1*], Ethan R. Deyle[1], Luis J. Gilarranz[2], and George Sugihara[1]

[1] Scripps Institution of Oceanography

University of California San Diego

La Jolla, CA, USA

[2] Integrative Ecology Group

Estación Biológica de Doñana, CSIC

Sevilla, Spain

[*] corresponding author

Hao Ye

9500 Gilman Drive MC0208

La Jolla, CA, USA 92093-0208

(858)-534-5582

hye@ucsd.edu

Model Systems with Random Coefficients

Two-Species Model System with Bidirectional Causality

We generalize the simple model system consisting of 2 coupled logistic difference equations as follows:

$$x(t+1) = x(t)\,[R_x - R_x\,x(t) - A_{xy}\,y(t)]$$
$$y(t+1) = y(t)\,[R_y - R_y\,y(t) - A_{yx}\,x(t\text{-}\tau_d)] \qquad \text{[Eq. S1]}$$

where τ_d is the time delay for the effect of x on y. For each simulation run, we sample a new fixed value for the growth rates R_x and R_y from the uniform distribution (3.7, 3.9), as well as new values for the interaction coefficients A_{xy} and A_{yx} from the uniform distribution (0.05, 0.1). In addition each simulation is initialized with random starting points with $x(1)$ and $y(1)$ drawn from the uniform distribution (0.01, 0.99), and run for 3000 time steps. For each of the different values for the time delay: $\tau_d = 0$, $\tau_d = 2$, and $\tau_d = 4$, we ran a total of 500 simulations (when populations reached negative values or increased beyond carrying capacity, we sampled new coefficients and re-ran the simulation). Using extended CCM, we analyze each simulation using $E = 2$, $\tau = 1$, selecting a random library of 200 vectors over time points 101-2000, and computing cross map skill for time points 2001-3000.

The results are depicted in Figure S1, with boxplots for the value of the cross map lag (l) that gives the highest cross map skill (ρ). Because nearly all simulations had identical values for the optimal cross map lag (l), the boxplots are depicted as straight lines with just a few outliers. As expected, "*y* xmap *x*" (red), depicting the causal effect of y on x has an optimal cross map lag of $l = -1$, because the y affects x with a lag of 1 time step ($y(t)$ influences x(t+1)). Conversely, the optimal cross map lag for "*x* xmap *y*" (blue) changes depending on τ_d; this is also expected since τ_d describes the time delay in the response of y to x. In fact, the optimal cross map lag for "*x*

xmap y" appears to accurately recover the time delay parameter τ_d: for example, the optimal l is nearly always -3 when $\tau_d = 2$ (meaning $x(t)$ influences $y(t+3)$ and therefore it takes 3 time steps for y to respond to x).

Two-Species Model System with Synchrony

We also generalize the modified form of the above system that produces synchrony with strong forcing from x to y only:

$$x(t+1) = x(t)\,[R_x - R_x\, x(t)]$$
$$y(t+1) = y(t)\,[R_y - R_y\, y(t) - A_{yx}\, x(t)] \qquad \text{[Eq. S2]}$$

For each simulation, R_x is sampled from the uniform distribution (3.7, 3.9), R_y is sampled from the uniform distribution (2.5, 3.2), and A_{yx} is sampled from the uniform distribution (0.7, 0.9). As above, the system is initialized with random starting points with $x(1)$ and $y(1)$ drawn from the uniform distribution (0.01, 0.99), and run for 3000 time steps. We ran a total of 500 simulations (when populations reached negative values or increased beyond carrying capacity, we sampled new coefficients and re-ran the simulation). Using extended CCM, we analyze each simulation using $E = 2$, $\tau = 1$, selecting a random library of 200 vectors over time points 101-2000, and computing cross map skill for time points 2001-3000.

Results for the "generalized synchrony" model are shown in Figure S2, with boxplots showing the value of the cross map lag (l) that gives the highest cross map skill (ρ). Again, we see that the optimal cross map lag (l) is generally negative in the direction of true causality (red, "y xmap x") and positive in the direction of synchrony (blue, "x xmap y").

Four-Species Model System

To test the robustness of extended CCM in distinguishing between direct and indirect causality, we generalize the 4-species model system with a transitive causal chain:

$$y_1(t+1) = y_1(t)\,[R_1 - R_1\,y_1(t)]$$

$$y_2(t+1) = y_2(t)\,[R_2 - A_{21}\,y_1(t) - R_2\,y_2(t)]$$

$$y_3(t+1) = y_3(t)\,[R_3 - A_{32}\,y_2(t) - R_3\,y_3(t)] \qquad \text{[Eq. S3]}$$

$$y_4(t+1) = y_4(t)\,[R_4 - A_{43}\,y_3(t) - R_4\,y_4(t)]$$

For each simulation, the growth parameters are sampled as follows: R_1 is drawn from the uniform distribution (3.8, 4.0), R_2 and R_3 are both drawn from the uniform distribution (3.5, 3.7), and R_4 is drawn from the uniform distribution (3.7, 3.9). The interaction parameters A_{21}, A_{32}, and A_{43} are all drawn from the uniform distribution (0.3, 0.5). As above, the system is initialized with random starting points with each $y_i(1)$ drawn from the uniform distribution (0.01, 0.99), and run for 3000 time steps. We ran a total of 500 simulations (when populations reached negative values or increased beyond carrying capacity, we sampled new coefficients and re-ran the simulation). Using extended CCM, we analyze each simulation using $E = 4$, $\tau = 1$, selecting a random library of 200 vectors over time points 101-2000, and computing cross map skill for time points 2001-3000.

Results for this analysis are shown in Figure S3, with bagplots (1) depicting the bivariate boxplots for the optimal cross map lags (l) and corresponding cross map skill (ρ). As in Figure 3, the top row of panel b shows that the optimal cross map lags are close to 0 and show high cross map skill, as would be expected for these direct interactions. In contrast, the indirect interactions generally have optimal cross map lags that are more negative, and lower cross map skill, with the most indirect interaction (from y_1 to y_4, identified using y_4 xmap y_1) showing the most negative cross map lag and the lowest cross map skill. We note that the variance in cross map skill is quite high, indicating that it may not be as useful in separating direct from indirect interactions in real systems, whereas cross map lag shows clearer separation.

References

1. Rousseeuw, P. J., Ruts, I., & Tukey, J.W. The Bagplot: A Bivariate Boxplot. *The American* Statistician **53** (4), 382–387 (1999).

Figures

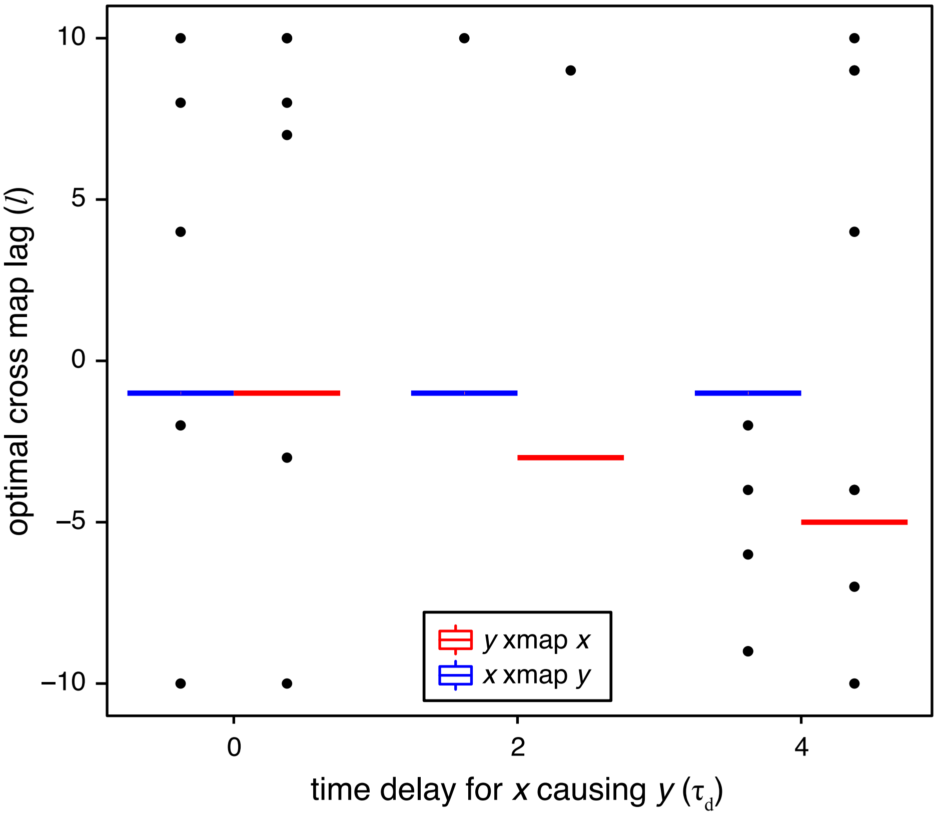

Figure 1 — Robustness of extended CCM in the 2-species logistic model with bidirectional forcing.

Boxplots of optimal cross map lag (l) are shown for 500 random simulations of the 2-species logistic model with bidirectional causality and with three different time delays, τ_d. Except for a few outliers, the optimal cross map lag when using x to cross map y (blue, "x xmap y") is -1, as would be expected, because x responds to y within a single time step. In the opposite direction, a larger time delay (τ_d) in the effect of x on y results in larger negative values for the optimal cross map lag when using y to cross map x (red, "y xmap x").

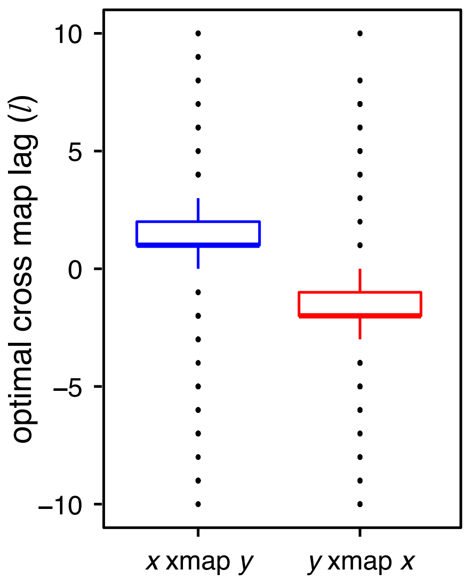

Figure 2 — Robustness of extended CCM in the 2-species logistic model with generalized synchrony.

Boxplots of optimal cross map lag (l) are shown for 500 random simulations of the 2-species logistic model with unidirectional causality producing generalized synchrony. Except for a few outliers, the optimal cross map lag when using y to cross map x (red, "y xmap x") is negative, and positive in the opposite direction (blue, "x xmap y"). This is expected, because x has a true causal influence on future values of y, mean y is better at cross mapping to past values of x; conversely, the lack of an actual effect of y on x, but rather "generalized synchrony" means that x is better at cross mapping future values of y.

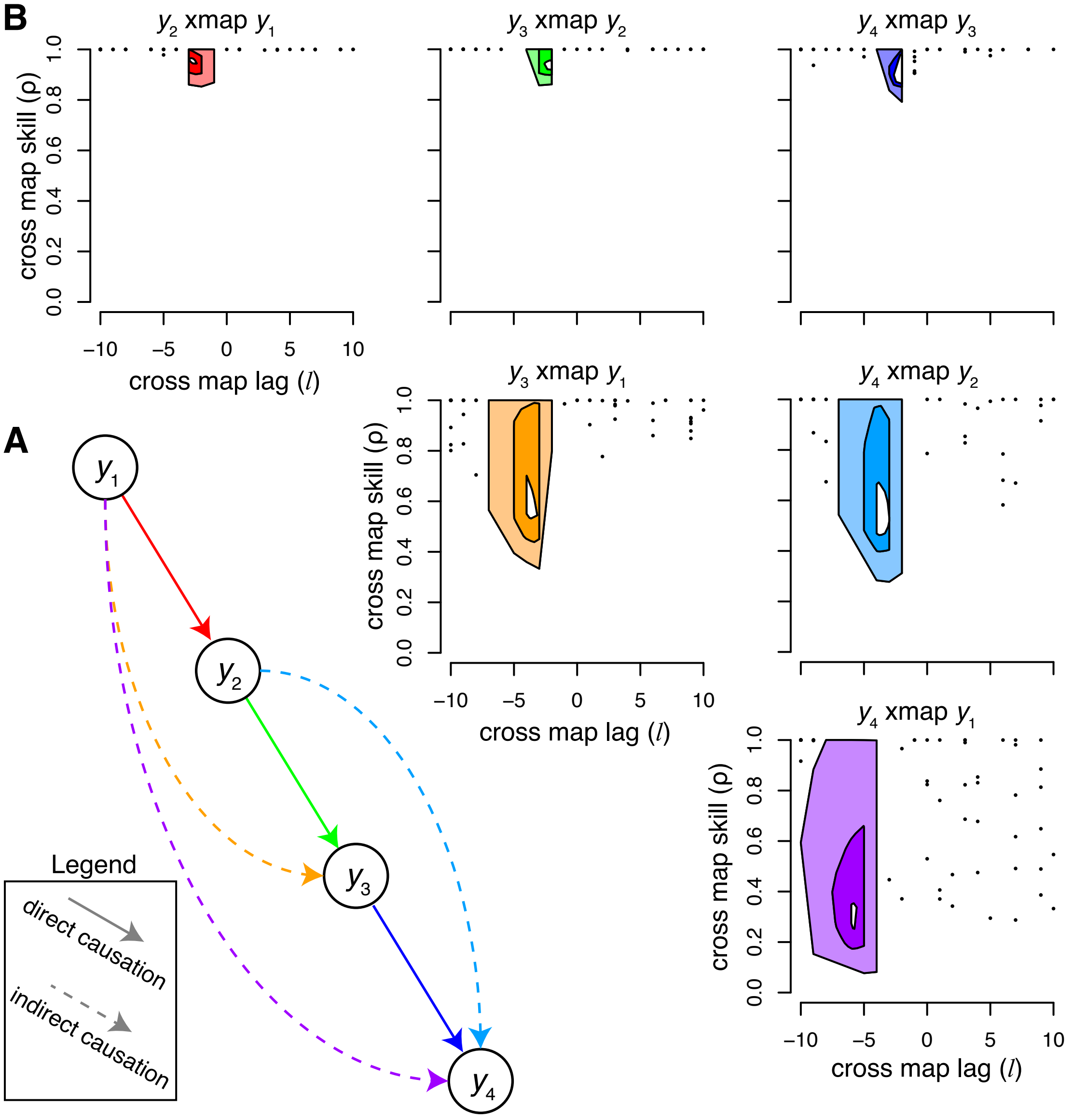

Figure 3 — Robustness of extended CCM for distinguishing direct and indirect causality in a transitive causal chain.

(**A**) In this system, y_1 causes y_2 causes y_3 causes y_4 such that indirect causation from y_1 to y_3, y_2 to y_4, and y_1 to y_4 occurs. (**B**) Bagplots show the optimal cross map lag (l) and corresponding cross map skill (ρ) for 500 random simulations of this system. The white central area depicts the 95% confidence interval for the median value, while the darker colored region is the "bag" containing the central 50% of points (i.e., similar to an interquartile range), and the lighter colored region is

the loop with area 3 times the size of the bag, as described in (1). The direct links (top row) are strongest with the highest cross map skill and the most immediate effects (l ~ -2), while the indirect links separated by one node (middle row) have moderate cross map skill and somewhat delayed effects (l ~ -4), and the indirect link from y_1 to y_4 (bottom row) is the weakest and with the longest time delay (l ~ -6).
